# Adiponectin Regulates Vascular Endothelial Growth Factor-C Expression in Macrophages via Syk-ERK Pathway

**DOI:** 10.1371/journal.pone.0056071

**Published:** 2013-02-12

**Authors:** Di Hu, Atsunori Fukuhara, Yugo Miyata, Chieko Yokoyama, Michio Otsuki, Shinji Kihara, Iichiro Shimomura

**Affiliations:** 1 Department of Metabolic Medicine, Osaka University Graduate School of Medicine, 2-2, Yamamdaoka, Suita, Osaka, Japan; 2 Department of Biomedical Informatics, Division of Health Sciences, Osaka University Graduate School of Medicine, 1–7, Yamamdaoka, Suita, Osaka, Japan; University College London, United Kingdom

## Abstract

Adiponectin is exclusively expressed in adipose tissues and exhibits protective effects against cardiovascular and metabolic diseases. It enhances AMP-activated kinase (AMPK) and peroxisome proliferator-activated receptor α (PPARα) signaling in the liver and skeletal muscles, however, its signaling pathways in macrophages remain to be elucidated. Here, we show that adiponectin upregulated the expression of vascular endothelial growth factor (VEGF)-C, and induced phosphorylation of extracellular signal-regulated kinase (ERK) in macrophages. Inhibition of Syk abrogated adiponectin-induced VEGF-C expression and ERK phosphorylation. Furthermore, inhibition of ERK blocked the induction of VEGF-C gene. Inhibition of Syk, but not that of ERK, abrogated adiponectin-induced expression of cyclooxygenase (COX)-2, tissue inhibitor of metalloproteinase (TIMP)-1, and interleukin (IL)-6. These results indicate that adiponectin regulates VEGF-C expression via Syk-ERK pathway in macrophages.

## Introduction

Recent studies have described the production and secretion of bioactive substances conceptualized as adipocytokines [e.g., leptin, adiponectin, tumor necrosis factor-α (TNF-α), and plasminogen activator inhibitor type-1 (PAI-1)] from adipose tissues [1]–[4]. We have also identified adiponectin as an adipocytokine from the human adipose tissue cDNA library [5]. Several studies have linked adiponectin to hypertension. For example, Iwashima et al. [6] reported significantly low adiponectin concentration in hypertensive patients, and that hypoadiponectinemia was an independent risk factor for hypertension. Furthermore, in the 5-year prospective study of Chow et al. [7], baseline serum adiponectin was reported as a significant independent predictor of hypertension. Based on these reports, hypoadiponectinemia is a suitable marker for predisposition to hypertension.

Machnik et al. [8] demonstrated the pathological role of vascular endothelial growth factor-C (VEGF-C) in salt-induced hypertension. High-salt diet leads to interstitial Na^+^ accumulation in the skin. In macrophages, high Na^+^ concentrations activate tonicity-responsive enhancer binding protein (TonEBP), which in turn induces the expression and secretion of VEGF-C. Furthermore, VEGF-C trapping by soluble VEGF receptor-3 results in elevation of blood pressure in response to high-salt diet.

We reported previously that adiponectin knock-out (KO) mice fed high-salt diet develop significant high systolic blood pressure compared with wild-type controls, without the appearance of insulin resistance [9], and that replenishment of adiponectin expression restored normal blood pressure. Accordingly, both adiponectin and VEGF-C are defense factors that can prevent salt-induced hypertension. To our knowledge, however, the relationship between adiponectin and VEGF-C has not been investigated. Here, we show for the first time that adiponectin is an inducer of VEGF-C in human and mouse macrophages. Furthermore, this induction is mediated through Syk-dependent ERK pathway.

## Materials and Methods

### Materials

Syk inhibitor (BAY 61-3606), Src inhibitor (PP1) and MEK inhibitor (PD98059) were purchased from Calbiochem (Gibbstown, NJ). Syk inhibitor (Piceatannol) was purchased from Tokyo Chemical Industry Co. (Tokyo, Japan). Anti-phosphorylated ERK1/2 (Thr202/Tyr204), and anti-ERK1/2 antibodies were purchased from Cell Signaling Technology (Danvers, MA). Protease and phosphatase inhibitor cocktail was from Pierce (Rockford, IL). VEGF-C concentrations of media were measured with enzyme-linked immunosorbent assay (ELISA) kit (R&D Systems, Minneapolis, MN). All other chemical reagents are purchased from Sigma-Aldrich (St Louis, MO).

### Cell Culture and Treatment

Human monocytes were isolated by density-gradient centrifugation, employing Lymphocyte Separation Medium (d = 1.077, Nacalai tesque, Kyoto, Japan) as described previously [9] and subsequence adherence to cell culture dishes from leukopacs of several healthy donors. Monocytes were cultured in RPMI-1640 medium (Invitrogen, Carlsbad, CA) containing 10% human serum (Gemini Bio-products, Calabasas, CA) for 7 days to obtain macrophages. Differentiated macrophages were incubated in medium RPMI-1640 containing 1% human serum with or without the indicated amount of insect cell-derived recombinant human adiponectin (Nosan Corp, Yokohama, Japan), or reagents for 6 hours.

RAW264.7 cells (American Type Culture Collection, Manassas, VA; ATCC no. TIB-71) were maintained in RPMI-1640 medium containing 10% fetal bovine serum (Eqvitech-Bio, Kerrville, TX). RAW264.7 cell were incubated in RPMI-1640 medium containing 1% fetal bovine serum (FBS) with or without the indicated amount of adiponectin or reagents for 6 hours.

### Quantitative Real-time PCR

Total RNA was extracted using RNA-STAT-60 (Tel-Test, Friendswood, TX) and the protocol supplied by the manufacturer. The cDNA was synthesized using the Transcriptor First Strand cDNA Synthesis Kit (Roche, Indianapolis, IL). Real-time PCR was performed on LightCycler system (FastStart DNA Master SYBR Green I, Roche) according to the protocol provided by the manufacturer. Table S1 lists the sequences of primers used for real-time PCR.

### Detection of ERK Phosphorylation

Macrophages were lysed in ice-cold lysis buffer (20 mM Tris/HCl, pH 7.2, 1 mM EGTA, 1% Triton X-100, 150 mM NaCl, and 100 mM NaF) containing a cocktail of protease and phosphatase inhibitors. The protein concentration of the lysates was determined by bicinchoninic acid assay (Pierce), followed by SDS-PAGE, and western blotting with anti-phosphorylated ERK antibody. Detection of the immune complex was carried out by ECL Advance Western Blot Detection System (GE Healthcare, Buckinghamshire, UK).

### Statistical Analysis

All data were expressed as mean ± SEM. Differences between two groups were examined for statistical significance by the Student’s t-test, and among four groups by one-way analysis of variance (ANOVA) followed by Tukey-Kramer test. A *P* value <0.05 denoted the presence of a statistically significant difference. The JMP Pro 9.0.3 software (SAS Institute. Inc., Cary, NC) was used in all statistical analyses.

## Results

### Adiponectin Induces VEGF-C Expression in Human Macrophages

**Figure 1:**
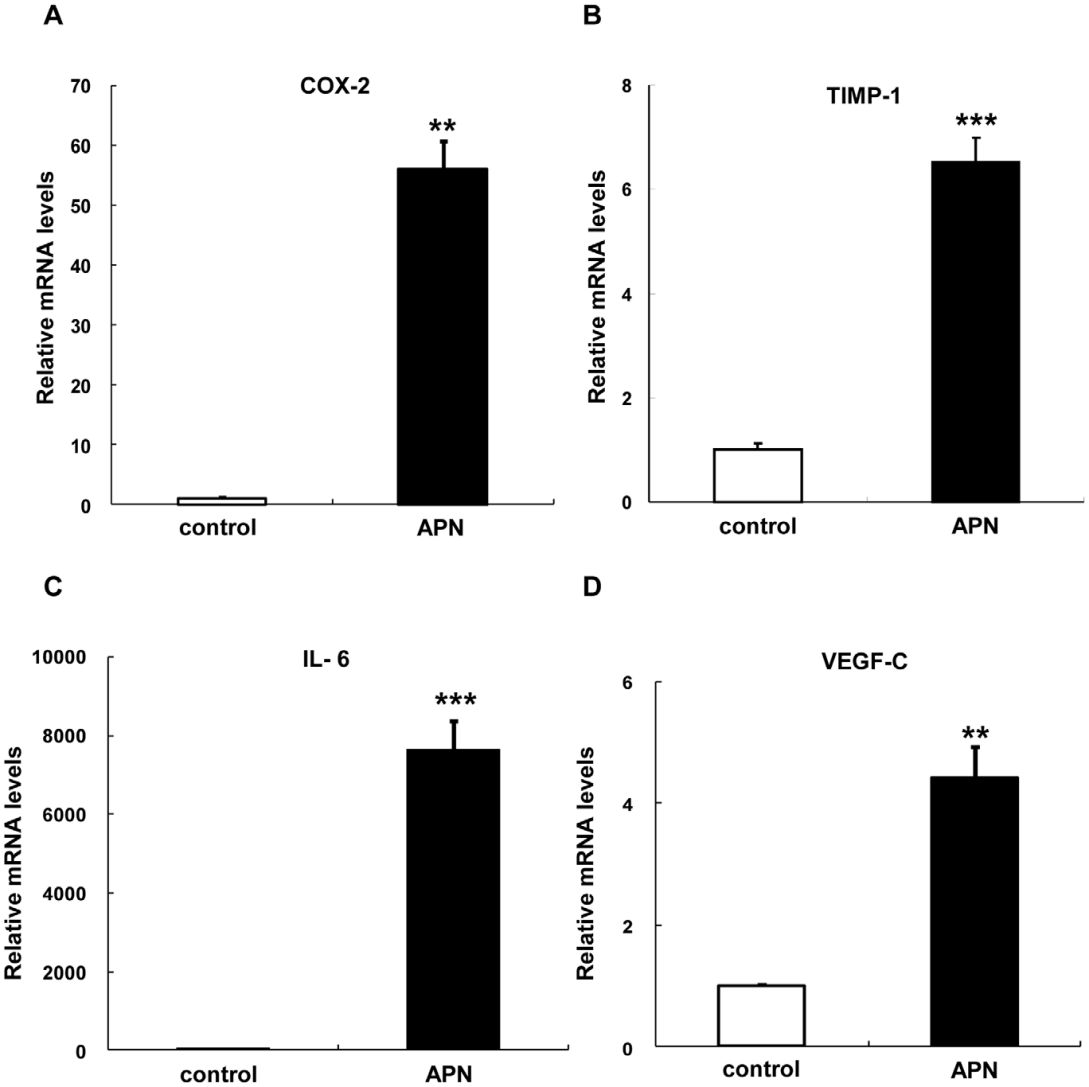
Effects of adiponectin on mRNA expression levels of various genes in peripheral blood monocyte-derived macrophages (PBDMs). After incubation for 7 days, mature human PBDMs were incubated for 6 hours with 10 µg/ml of adiponectin protein. The mRNA expression levels of COX-2 (A), TIMP-1 (B), IL-6 (C), and VEGF-C (D) were quantified by real-time PCR. Values are normalized to the level of GAPDH mRNA and expressed as mean ± SEM (n = 3). **P<0.01, ***P<0.001.

To examine the effects of adiponectin on gene expression levels, human peripheral blood monocyte*-*derived macrophages (PBDMs) were incubated with insect cell-derived recombinant human adiponectin. As described previously [10]–[12], we confirmed that treatment with adiponectin induced the expression of cyclooxygenase-2 (COX-2) (Fig. 1A), tissue inhibitor of metalloproteinase (TIMP)-1 (Fig. 1B), and IL-6 genes (Fig. 1C). We also confirmed that treatment with adiponectin reduced the expression levels of type I class A macrophage scavenger receptor (SRAI) [13], and increased those of ATP*-*binding cassette transporter A1 (ABCA1), and liver X receptor (LXR) α [14] (data not shown). Under the experimental conditions, adiponectin significantly induced the expression of VEGF-C mRNA (Fig. 1D). We next investigated the VEGF-C protein levels secreted into the media. Adiponectin also augmented secreted levels of VEGF-C (Fig. S1).

The next experiment determined that the above effects were not specific to human macrophages. Adiponectin induced the expression of COX-2 [10] (Fig. S2A), and IL-6 [12] (Fig. S2B) mRNAs in RAW264.7 murine macrophages. Furthermore, we also confirmed that adiponectin induced VEGF-C mRNA expression levels in the same murine macrophages (Fig. S2C).

### Adiponectin-induced Phosphorylation of ERK in PBDMs

**Figure 2:**
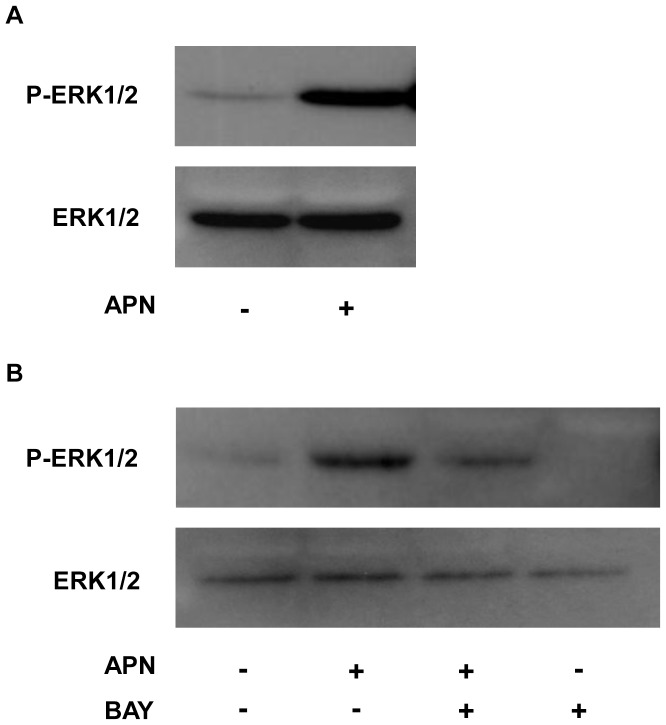
Adiponectin-induced phosphorylation of ERK in peripheral blood monocyte-derived macrophages (PBDMs). PBDMs were incubated for 20 min with 10 µg/ml of adiponectin protein. Cell lysates were subjected to SDS-PAGE followed by western blotting with anti-phosphorylated ERK1/2, and anti-ERK1/2 antibodies (A). PBDMs were preincubated with 10 µM BAY 61-3606 (BAY) for 30 min and then treated for 20 min with 10 µg/ml of adiponectin protein. Cell lysates were subjected to SDS-PAGE followed by western blotting with anti-phosphorylated ERK1/2, and anti-ERK1/2 antibodies (B). Equal protein loading and transfer were confirmed with Ponceau staining.

Previous reports showed that adiponectin induces ERK phosphorylation in beta cells and keratinocytes [15], [16], and also Syk tyrosine kinase activation in platelets [17]. In this regard, Syk is known to regulate the expression of VEGF-C [18]. Based on this background, we focused in the next experiment on Syk and ERK. Adiponectin increased the phosphorylation of ERK1/2 (Fig. 2A), similar to beta cells and keratinocytes. In dendritic cells, ERK is a signaling intermediate downstream of Syk. Furthermore, zymosan-induced activation of ERK is blocked in Syk-deficient dendritic cells [19]. To further clarify the signaling pathway in the response to adiponectin, we used BAY 61-3606 (BAY), a highly specific inhibitor of Syk tyrosine kinase, in combination with adiponectin. Pretreatment with BAY suppressed adiponectin-induced phosphorylation of ERK1/2 (Fig. 2B). These results indicate that adiponectin induces Syk-dependent ERK activation in macrophages.

### Effect of Syk Inhibitors on Adiponectin-mediated mRNA Induction

**Figure 3:**
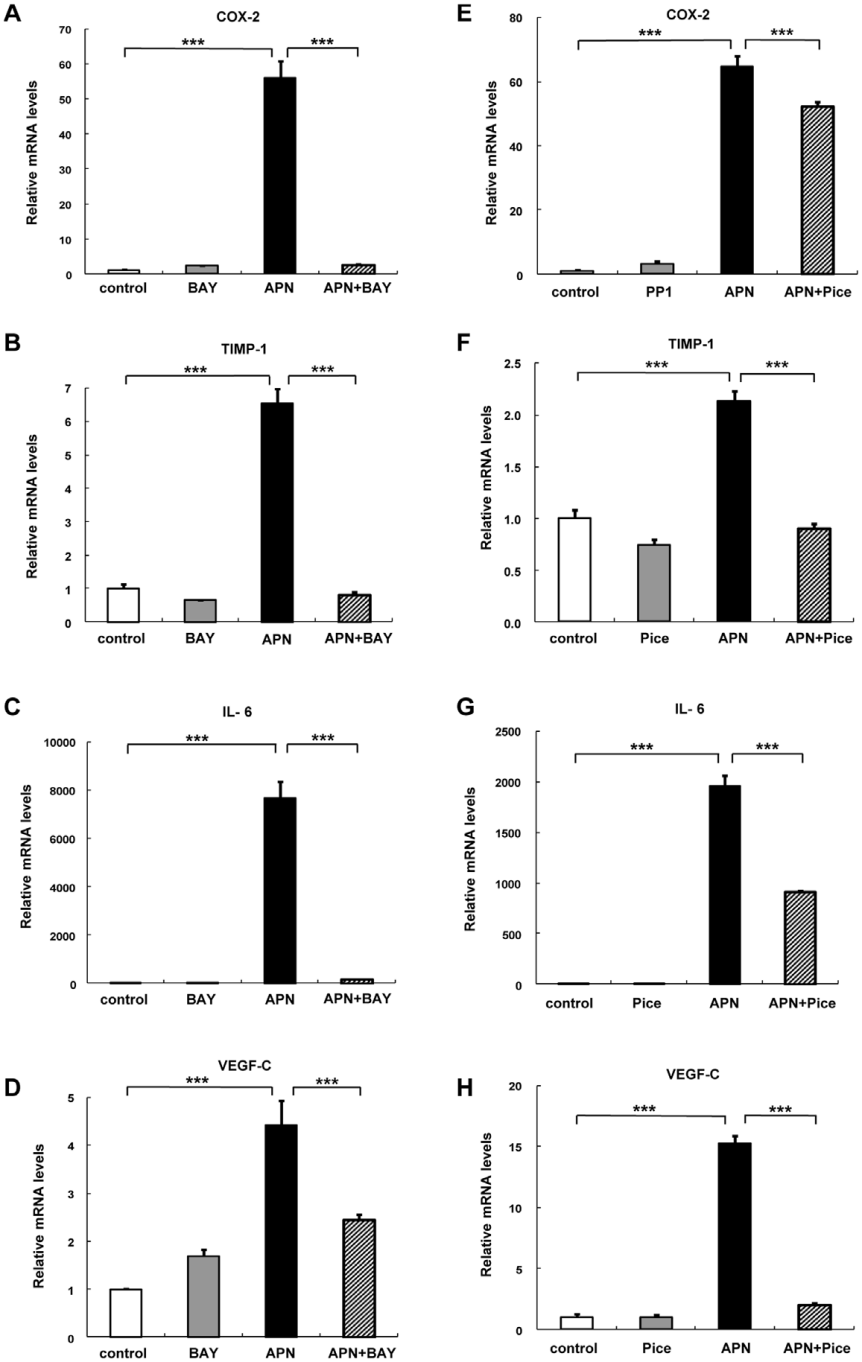
Effects of Syk inhibitors on the adiponectin-induced changes in peripheral blood monocyte-derived macrophages (PBDMs). PBDMs were preincubated with 10 µM BAY 61-3606 (BAY) (A, B, C, D), or 100 µM piceatannol (E, F, G, H) for 30 min followed by treatment with 10 µg/ml of adiponectin for 6 hours. The mRNA expression levels of COX-2 (A, E), TIMP-1 (B, F), IL-6 (C, G), and VEGF-C (D, H) were quantified by real-time PCR. Values are normalized to the level of GAPDH mRNA and expressed as mean ± SEM (n = 3). ***P<0.001.

To determine the role of kinase signaling in adiponectin-mediated mRNA induction, PBDMs were treated with Syk inhibitors. Induction of COX-2, TIMP-1, IL-6, and VEGF-C by adiponectin was almost completely blocked by BAY (Fig. 3A–D). Moreover, piceatannol, another Syk inhibitor, significantly reduced adiponectin-mediated induction of these genes (Fig. 3E–H). Adiponectin-mediated induction of COX2, IL-6, and VEGF-C was also significantly reduced in RAW264.7 macrophages (Fig. S3A–C), confirming that the above effects were not specific to human macrophages. Considered together, these results indicate the involvement of the Syk pathway in adiponectin-mediated induction of the aforementioned factors in macrophages.

In hepatocytes, adiponectin enhances fatty acid oxidation through activation of AMP-activated kinase (AMPK) and peroxisome proliferator-activated receptor α (PPARα) [20]. To examine the roles of these signaling in the induction of VEGF-C mRNA, PBDMs were treated with AICAR, an AMPK activator, and fenofibrate, a PPAR-α activator. Treatment with AICAR had no effect on VEGF-C expression (data not shown). On the other hand, fenofibrate significantly increased VEGF-C mRNA expression level by 1.3 times compared to the control (data not shown). However, treatment with GW6471, a specific inhibitor of PPAR-α, did not change adiponectin-mediated induction of VEGF-C mRNA expression (data not shown).

### Effects of Inhibitors of Src-family Kinase and ERK on Adiponectin-mediated mRNA Induction

**Figure 4:**
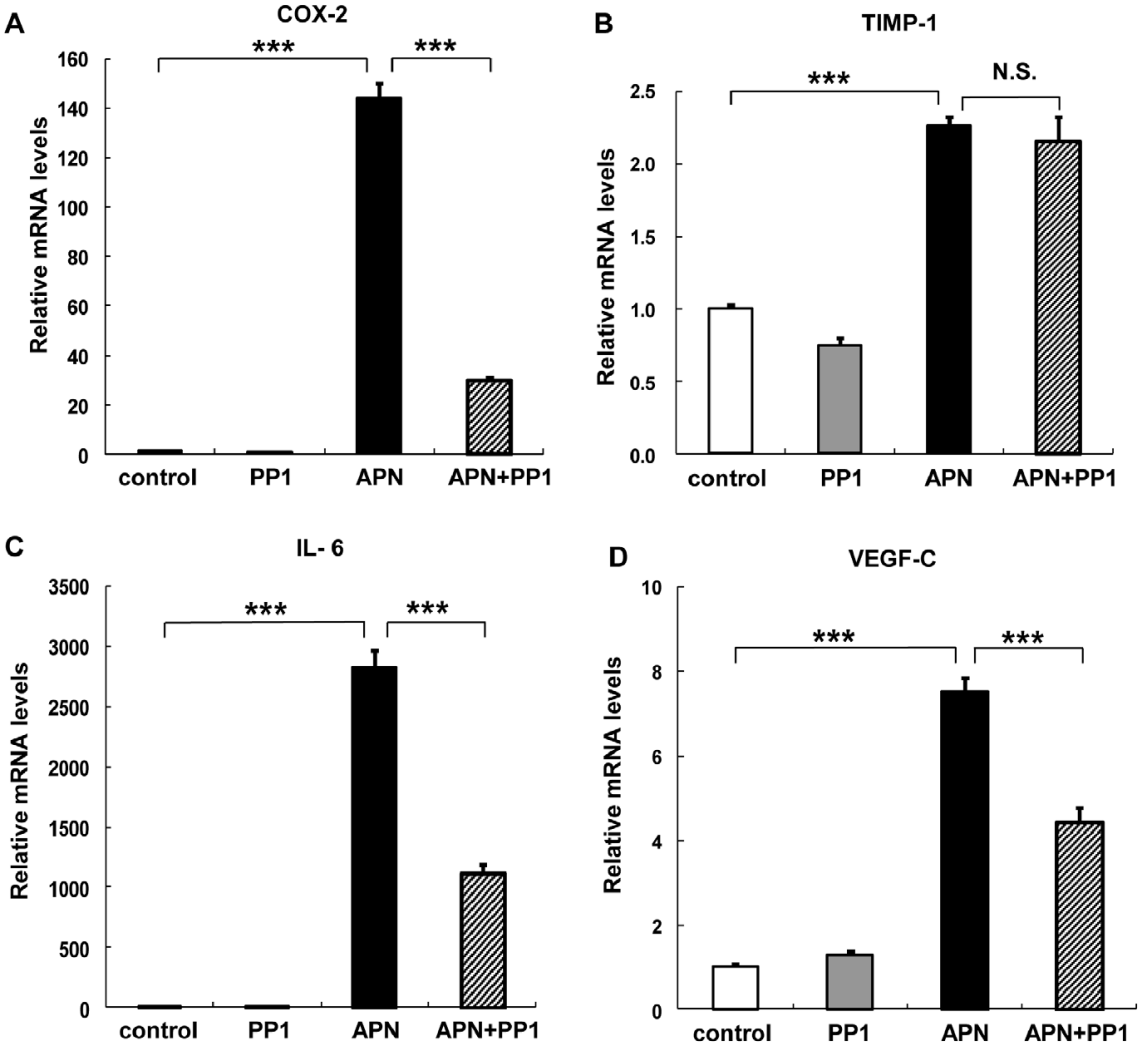
Effects of Src inhibitor on adiponectin-induced changes in peripheral blood monocyte-derived macrophages (PBDMs). PBDMs were preincubated with 10 µM PP1 for 30 min and then incubated with adiponectin for 6 hours (A–D). The mRNA expression levels of COX-2 (A), TIMP-1 (B), IL-6 (C), and VEGF-C (D) were quantified by real-time PCR. Values are normalized to the level of GAPDH mRNA and expressed as mean ± SEM (n = 3). ***P<0.001. N.S not significant.

**Figure 5:**
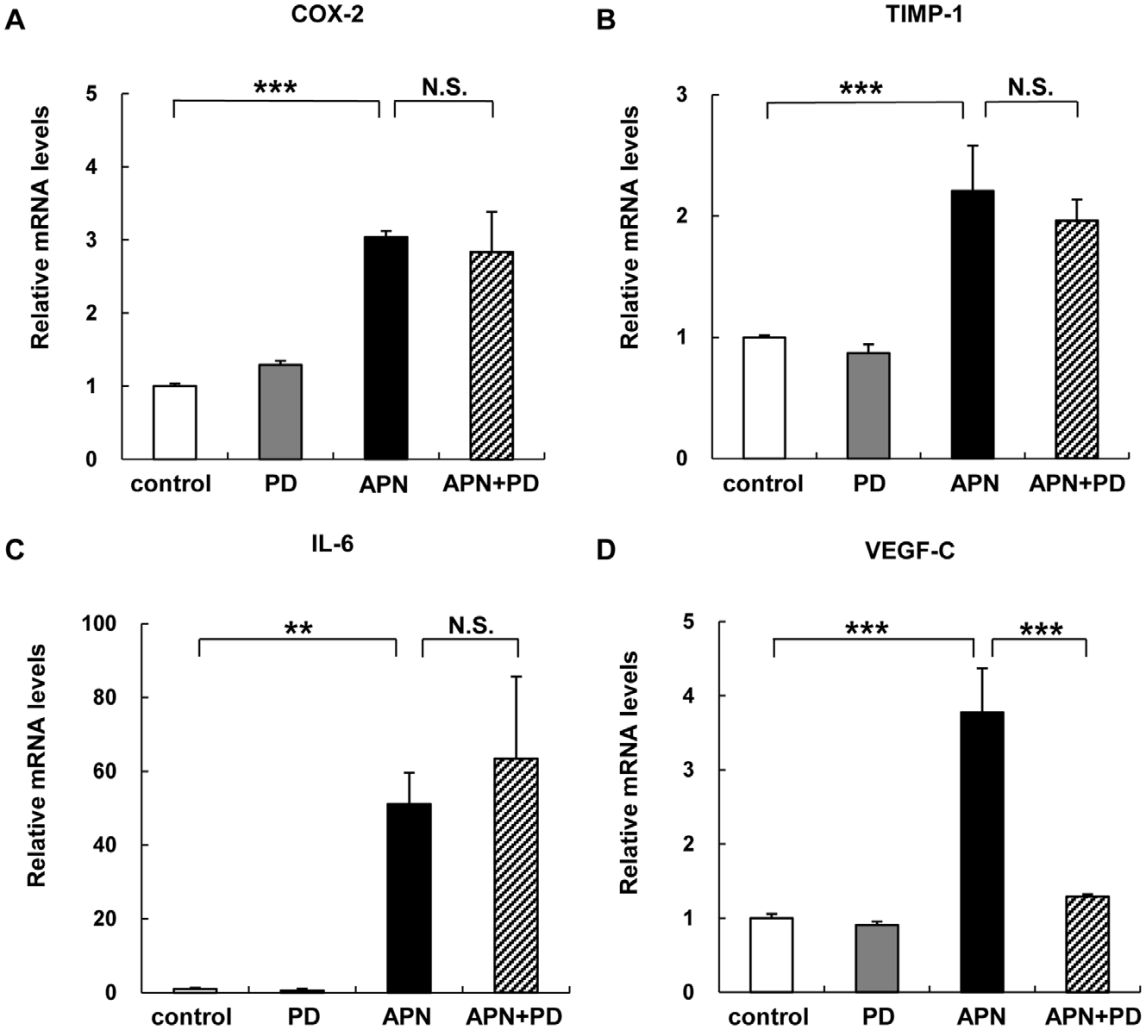
Effects of MEK inhibitor on adiponectin-induced changes in PBDMs. PBDMs were preincubated with 50 µM PD98059 followed by treatment with adiponectin for 6 hours (A-D). The mRNA expression levels of COX-2 (A), TIMP-1 (B), IL-6 (C), or VEGF-C (D) were quantified by real-time PCR. Values are normalized to the level of GAPDH mRNA and expressed as mean ± SEM (n = 3). ***P<0.001. N.S not significant.

Previous reports showed that Src family kinases are upstream signaling molecules of Syk, and that B-cell antigen receptor-mediated activation of Src family kinases results in phosphorylation of Syk in immunocytes [21]. In the next series of experiment designed to investigate the effect of Src family kinase on the expression of adiponectin-regulated genes, treatment of PBDMs with PP1, a Src-family kinase inhibitor, abrogated adiponectin–induced expression of COX-2, IL-6, and VEGF-C, but not TIMP-1 (Fig. 4A–D). Finally, we addressed the significance of ERK. Treatment of PBDMs with PD98059, a MEK 1/2 inhibitor, abrogated adiponectin–induced expression of VEGF-C, but not that of COX-2, TIMP-1, and IL-6 (Fig. 5A–D). Taken together, the results indicate that activation of both Src and ERK regulates VEGF-C expression.

## Discussion

Adiponectin has anti-inflammatory properties in macrophages, and modulates the expression levels of several cytokines. Treatment of cultured macrophages with adiponectin significantly inhibited their phagocytic activity and their lipopolysaccharide (LPS) -induced production of tumor necrosis factor-α [22]. Furthermore, adiponectin induced the expression and secretion of IL-10, which has anti-inflammatory function [11]. Okamoto et al. [23] used LPS-stimulated human macrophages to demonstrate the inhibitory effects of adiponectin on the expression of T-lymphocyte chemoattractants, such as interferon-inducible protein 10 (IP-10), IFN-inducible T-cell α-chemoattractant (I-TAC), and MIG (monokine induced by IFN-γ). In addition to the immunoregulatory properties, adiponectin is also reported to enhance insulin sensitivity by increasing hepatic IRS-2 expression via macrophage-derived IL-6-dependent pathway [12]. The present study added another action to adiponectin; we showed for the first time that adiponectin activates VEGF-C gene in macrophages.

Machnik et al. [8] reported that VEGF-C is responsible for the clearance of interstitial hypertonic volume retention, and serve as a defense against hypertension in response to high-salt diet, and our group reported that adiponectin KO mice develop high systolic blood pressure in response to high-salt diet [9]. In the present study, we showed for the first time that adiponectin is an inducer of VEGF-C in macrophages. Taken together, anti-hypertensive effect of adiponectin may be partly accounted for by its induction of VEGF-C, which possibility remains to be elucidated.

In addition to macrophages, VEGF-C mRNA expression has been demonstrated in a large number of human tumors, such as breast, colon, lung, thyroid, gastric cancers and melanomas. VEGF-C overexpression in breast cancer cells potently increased intratumoral lymphangiogenesis, resulting in significantly enhanced metastasis to regional lymph nodes and to lungs, suggesting VEGF-C as a molecular link between tumor lymphangiogenesis and metastasis [24]. On the other hand, adiponectin prevents new blood vessel growth in chorioallantoic membrane and corneal angiogenesis assays. In a mouse tumor model, adiponectin inhibits primary tumor growth, and this effect is associated with decreased neovascularization, and increased tumor cell apoptosis [25]. Further investigation should be required to reveal the effect of adiponectin on VEGF-C expression in cancer cells.

Many of the actions of adiponectin in hepatocytes and myocytes are attributed to the activation of AMPK and PPARα [20]. On the other hand, our data showed that AICAR, an AMPK activator, did not induce VEGF-C expression. Fenofibrate, a PPARα ligand, significantly induced VEGF-C expression, however, the induction ratio was relatively small (1.3 times compared to control), and inhibition of PPARα did not reduce adiponectin-induced VEGF-C expression. Recent studies have indicated that VEGF-C expression is regulated by Syk [18] and that adiponectin activates Syk in platelets [17]. Syk is highly expressed in hematopoietic cells, where it is known to play a crucial role in adaptive immune receptor signaling. Furthermore, Syk-expressing myeloid cells are reported to produce various angiogenic factors, such as VEGF-C, growth factors and chemokines [26]. Based on these reports, macrophages were treated in the present study with inhibitors of Syk, Src and ERK. The results showed that both Syk and Src regulated the majority of the known target genes of adiponectin in macrophages. Since adiponectin induces ERK phosphorylation in a Syk-dependent manner, it is reasonable to conclude that Syk plays a major role in adiponectin signaling.

Adiponectin binds to several types of receptors depending on the cell type. In hepatocytes and myocytes, adipoR1 and adipoR2 serve as receptors for adiponectin, which upon binding induce glucose utilization, fatty acid oxidation and mitochondria genesis [20]. In platelets, glycoprotein (GP) VI acts as a receptor for the globular domain of adiponectin, and induces platelet aggregation through Syk phosphorylation [17]. In macrophages, the globular domain of adiponectin predominantly binds to the AdipoR1 receptor and suppresses TLR-mediated nuclear factor (NF)-κB signaling [27]. On the other hand, Awazawa et al. [12] reported that adiponectin induces IL-6 production by macrophages independent of AdipoR1 and AdipoR2. Furthermore, another group reported that adiponectin binds to calreticulin on the macrophage cell surface, and promotes the clearance of early apoptotic cells [28].

We performed semiquantitative RT-PCR analysis of adiponectin receptors, and transcripts of AdipoR1, AdipoR2, calreticulin, and GPVI were detected (data not shown). Consequently, we suppose that these four proteins may be candidates of adiponectin receptor in macrophage. Further study is required to reveal the adiponectin receptor responsible for syk activation.

In conclusion, the present study demonstrated that adiponectin regulates the production of VEGF-C in macrophages via Syk-ERK pathway.

## Supporting Information

Figure S1 **Effects of adiponectin on protein levels in PBDMs.** PBDMs were incubated for 6 or 24 hours with 10 µg/ml of adiponectin protein. VEGF-C concentrations in media were measured by ELISA. Values expressed as mean ± SEM (n = 3). ***P<0.001.
(PDF)

Figure S2 **Effects of adiponectin on mRNA expression levels in murine RAW264.7 macrophages.** RAW264.7 macrophages were incubated for 6 hours with 10 µg/ml of adiponectin protein. The mRNA expression levels of COX-2 (A), IL-6 (B), and VEGF-C (C) were quantified by real-time PCR. Values are normalized to the level of 36B4 mRNA and expressed as mean ± SEM (n = 3). ***P<0.001.
(PDF)

Figure S3 **Effects of syk inhibitor on adiponectin-induced changes in murine RAW264.7 macrophages.** RAW264.7 macrophages were incubated with adiponectin and 10 µM BAY 61-3606 (BAY) for 6 hours. The mRNA expression levels of COX-2 (A), IL-6 (B), and VEGF-C (C) were quantified by real-time PCR. Values are normalized to the level of 36B4 mRNA and expressed as mean ± SEM (n = 3). ***P<0.001.
(PDF)

Table S1 (DOC)
