# Supplementary figures and images for: Adiponectin Regulates Vascular Endothelial Growth Factor-C Expression in Macrophages via Syk-ERK Pathway
Source: PLoS One. 2013 Feb 12;8(2):e56071. doi: 10.1371/journal.pone.0056071 (PMC3570530; doi:10.1371/journal.pone.0056071)

Figure S1.

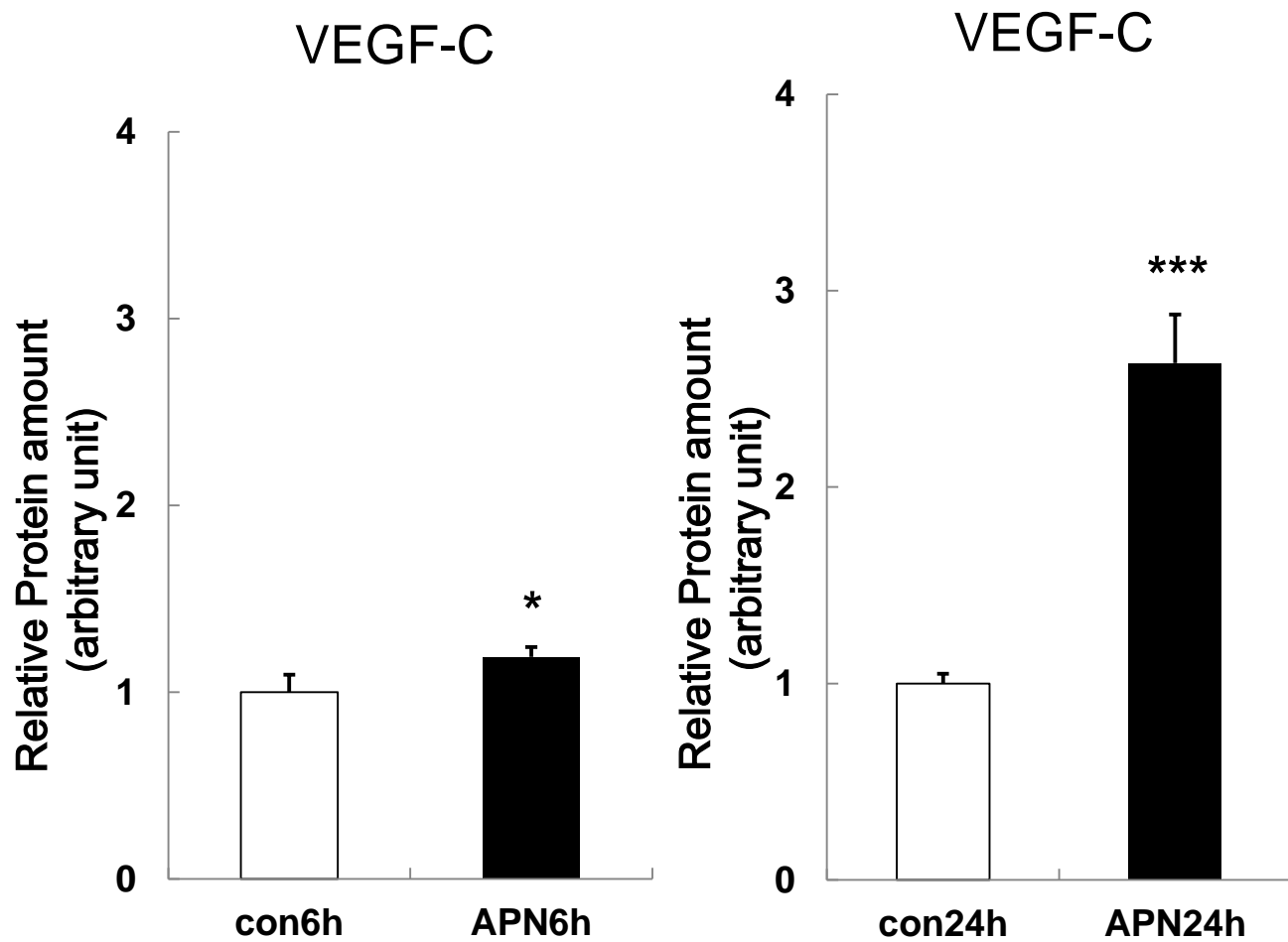

Supplement: Figure S1 — Effects of adiponectin on protein levels in PBDMs. PBDMs were incubated for 6 or 24 hours with 10 µg/ml of adiponectin protein. VEGF-C concentrations in media were measured by ELISA. Values expressed as mean ± SEM (n = 3). ***P<0.001. (PDF) [file pone.0056071.s001.pdf]

Figure S2.

A

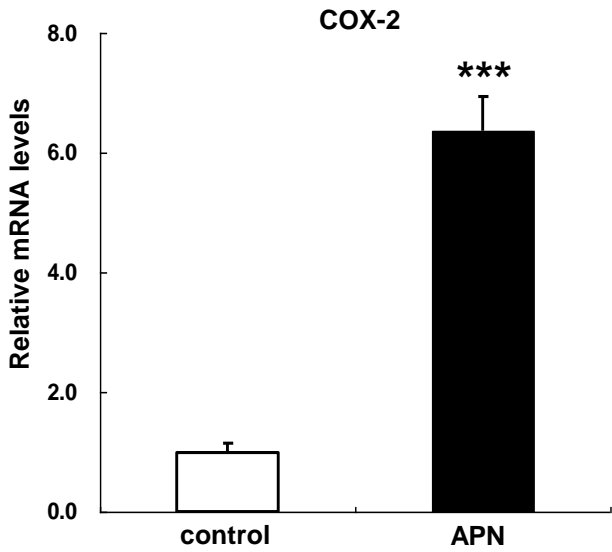

B

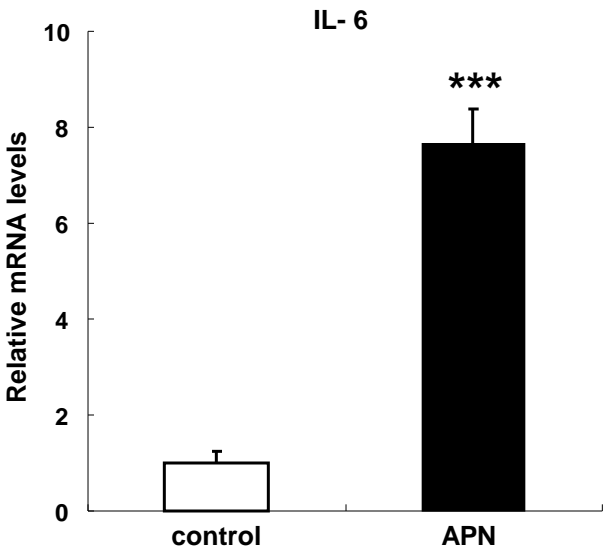

C

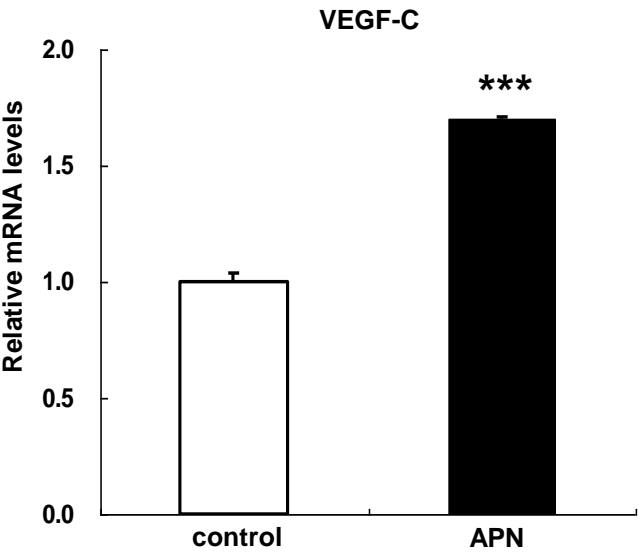

Supplement: Figure S2 — Effects of adiponectin on mRNA expression levels in murine RAW264.7 macrophages. RAW264.7 macrophages were incubated for 6 hours with 10 µg/ml of adiponectin protein. The mRNA expression levels of COX-2 (A), IL-6 (B), and VEGF-C (C) were quantified by real-time PCR. Values are normalized to the level of 36B4 mRNA and expressed as mean ± SEM (n = 3). ***P<0.001. (PDF) [file pone.0056071.s002.pdf]

Figure S3.

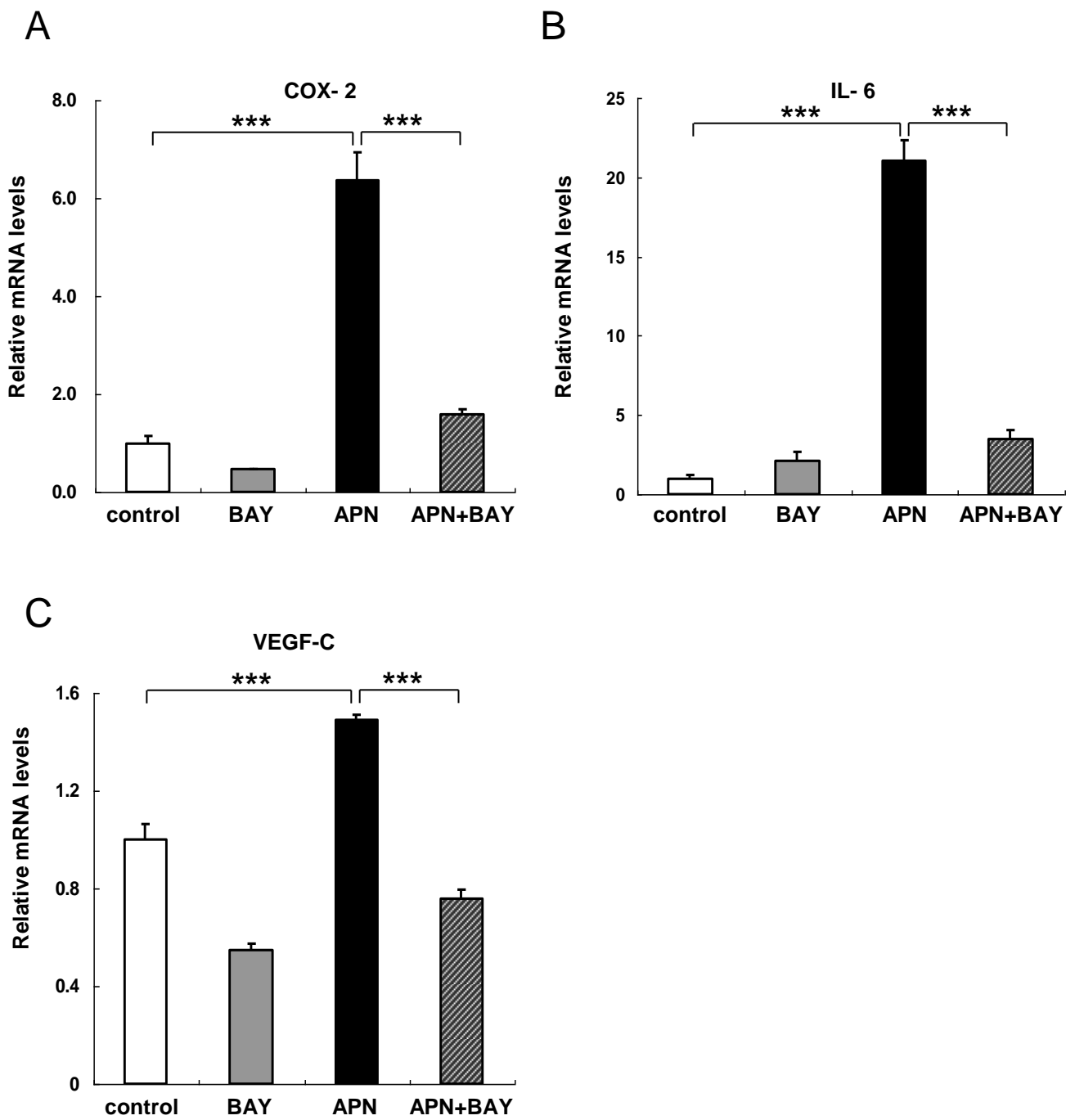

Supplement: Figure S3 — Effects of syk inhibitor on adiponectin-induced changes in murine RAW264.7 macrophages. RAW264.7 macrophages were incubated with adiponectin and 10 µM BAY 61-3606 (BAY) for 6 hours. The mRNA expression levels of COX-2 (A), IL-6 (B), and VEGF-C (C) were quantified by real-time PCR. Values are normalized to the level of 36B4 mRNA and expressed as mean ± SEM (n = 3). ***P<0.001. (PDF) [file pone.0056071.s003.pdf]
