# Supplementary material for: Adiponectin Regulates Vascular Endothelial Growth Factor-C Expression in Macrophages via Syk-ERK Pathway
Source: PLoS One. 2013 Feb 12;8(2):e56071. doi: 10.1371/journal.pone.0056071 (PMC3570530; doi:10.1371/journal.pone.0056071)
Supplement: Table S1 — (DOC) [file pone.0056071.s004.doc]

**Table S1.**

| Primer used for real-time RT-PCR (human) | |
| --- | --- |
| GAPDH  COX-2  TIMP-1  IL-6  VEGF-C | Forward 　5'-CCTGTTCGACAGTCAGCCG-3'  Reverse　 5'-CGACCAAATCCGTTGACTCC-3'  Forward　　5'-TCATCTGCAATAACGTGAAGGGC-3'  Reverse　　5'-GGAGCGGGAAGAACTTGCATT-3'  Forward　　5'-CCTGTTGTTGCTGTGGCTGA-3'  Reverse　　5'-CATAACGCTGGTATAAGGTGGTCTG-3'  Forward　　5'-AAATTCGGTACATCCTCGACGG-3'  Reverse　　5'-GGAAGGTTCAGGTTGTTTTCTGC-3'  Forward　　5'-TGCCAGCAACACTACCACAG-3'  Reverse　　5'-GTGATTATTCCACATGTAATTGGTG-3' |

| Primer used for real-time RT-PCR (mouse) | |
| --- | --- |
| 36B4  COX-2  IL-6  VEGF-C | Forward 　5'-GCTCCAAGCAGATGCAGCA-3'  Reverse　 5'-CCGGATGTGAGGCAGCAG-3'  Forward　　5'-CTGTACCCGGACTGGATTCT-3'  Reverse　　5'-TCCAGACTCCCTTGAAGTGG-3'  Forward　　5'-ACAACCACGGCCTTCCCTACTT-3'  Reverse　　5'-CACGATTTCCCAGAGAACATGTG-3'  Forward　　5'-AGATCCTGA Hphagethe mRNA level s000000000000000000000000000000000000000000000000000000000000000000000000000000000000000000000000000000AAAGTATTGATAATGAGTGG-3'  Reverse　　5'-CGTGGCATGCATTGAGTCTT-3' |
